# Appendix on Alcohol

**Published:** 1828-05

**Authors:** Daniel Drake


### APPENDIX.

### ALCOHOL.

Alcohol, according to Saussáre, is composed of Carbon, Oxygen and Hydroden, in the following proportions:—C. 51.98. O. 34.32. H. 13.70—100. Its specific gravity is 791 water being 1000; but it can seldom be obtained so pure, and its more common s. g. is 820. It was once supposed that alcohol was formed in the process of *distillation;* but Mr. Brande has demonstrated that it is exclusively the product of *fermentation;* and, therefore, that it exists in every sacharine or sugary solution, which has undergone fermentation. Sugar is indeed the indispensable material out of which alcohol is formed; and it is melancholy to reflect on the misapplication of art, in converting one of the most pleasant, harmless and nourishing substances, in nature, into a bewitching poison.

The following table, compiled from Brande's Manual of chemistry, shews the proportion in which alcohol exists in several different beverages:—

| | | |
|---|---|---|
| DISTILLED SPIRITS. | | |
| Scotch Whiskey - | 54.32 | parts by measure in 100 |
| Irish do. - - | 53.90 | do. |
| Rum - - - - | 53.68 | do. |
| Brandy - - | 53.39 | do. |
| Gin - - - - - | 51.60 | do. |
| WINES. | | |
| Port - - - - | 22.96 | do. |
| Madeira - - - | 22.27 | do. |
| Currant - - - | 20.55 | do. |
| Teneriffe - - - | 19.79 | do. |

| | | |
|---|---|---|
| Sherry - - - | 19.17 | do. |
| Lisbon and Malaga, each | 18.94 | do. |
| Claret - - - | 15.10 | do. |
| Champaign - - | 13.80 | do. |
| Gooseberry - - | 11.84 | do. |
| Elder - - - - | 8.79 | do. |
| MALT LIQUORS. | | |
| Ale - - - - | 6.87 | do. |
| Brown Stout - - | 6.80 | do. |
| London Porter - - | 4.20 | do. |
| London Small Beer - | 1.28 | do. |
| CIDER. | | |
| Highest average - - | 9.87 | do. |
| Lowest average - - | 5.21 | do. |

From this table it appears that in Brandy, Rum and Whiskey there is, by measure, more alcohol than water, and that Madeira Wine and Port Wine contain nearly half, strong Cider about a fifth, and Ale an eighth as much as they. Thus a botttle of Madeira has in it nearly a pint of proof spirits; a quart of Strong Cider more than six ounces; and a bottle of Ale about four ounces.

The chemists were surprised by the results of Mr. Brande's experiments. Nobody, till then, was aware, that the various fermented liquors contain so large a quantity of alcohol. Their intoxicating effects are certainly not in proportion. This arises from their other ingredients; which give to all of them a nourishing quality, and, to each, effects more or less peculiar. Thus, says Dr. Paris—

"Alcohol, although diluted to the degree of proof spirit, is still too strong for internal exhibition; indeed, where its use is indicated, it is more generally given in the form of wine, malt liquors, or ardent spirits, which must be regarded only as diluted alcohol, although each has a peculiarity of operation, owing to the modifying influence of the other elements of the liquid; thus *Brandy* is said to be simply cordial and stomachic; *Rum*, heating and sudorific; *Gin* and *Whiskey*, diuretic; and *Arrack*, styptic, heating and narcotic; it seems also probable that a modified effect is produced by the addition of various other substances, such as sugar and acids, which latter bodies, besides their anti narcotic powers, appear to act by favouring a more perfect combination and mutual penetration of the particles of spirit and water. The effects also which are produced by the habitual use of fermented liquors, differ essentially, according to the kind that is drank; thus Ale and Porter, in consequence of the nutritive matter, and perhaps the invigorating bitter with which they are charged, and the comparatively small proportion of alcohol which they contain, dispose to a plethora, which is not unfrequently terminated by appoplexy; Spirits on the other hand, induce severe dyspepsia, obstructed and hardened liver, dropsy, and more than half of all our chronical diseases; and Dr. Darwin moreover remarks that when they arise from this cause, they are liable to become hereditary, even to the third generation, gradually increasing, if the cause be continued, till the family becomes extinct: with regard to wine, Rush has truly observed that its effects, like those of tyranny in a well formed government, are first felt in the extremities, while spirits, like a bold invader, seize at once upon the vitals of the constitution; the different kinds of wine, however, produce very different and even opposite effects." Pharmacologia, pp. 377—8

This view of the effects of different kinds of alcoholic beverages, suggests to us the propriety of nice discrimination, when we are about to choose one of them as a medicine. On the subject of their relative differences, both physicians and the people, are less informed than they ought to be, and mistakes are, therefore, by no means uncommon.

In their praiseworthy zeal for the suppression of intemperance, many persons have proposed the enactment of a law, prohibiting the distillation of alcohol. But such a law would not be compatible with the genius of a free government; and could never be successfully enforced. Alcohol, moreover, is an agent of extensive and varied utility, and could not, therefore, be dispensed with. In analytical chemistry, it has contributed much to a correct knowledge of the composition of bodies; especially those belonging to the vegetable kingdom. In the arts it performs a service equally important, being the only solvent for a great number of substances which, in civilized life, are highly useful, if dissolved in alcohol; but otherwise useless. Lastly, in medicine its uses are equally important; as it extracts the active principles from a number of native vegetable compounds, and imparts to them increased energy as tonics and stimulants.

If, however, it were practicable and proper, to suppress the distillation of alcohol, but little would perhaps be gained by so doing. An increased manufacture and consumption of fermented liquors would no doubt ensue; or opium might be introduced, as a substitute, the effects of which would not be less dreadful than those of ardent spirits. Distillation, therefore, must be tolerated, but it should be taxed. This would diminish the quantity of its products; and raise their price to the consumer, which would infallibly diminish their consumption. It is said, however, that the people will not submit to an excise. If this be the fact, it argues but little in favor of their temperance, or their regard for the interests of morality. The people may not submit to an excise unaccompanied by an increased duty on foreign spirits and wines; but with such a duty,—which would keep the rich and poor in the same *relative* condition, by augmenting, equally, to all the expense of drinking,—an excise would probably be borne, and could not fail to produce salutary effects.

